# Unmet Needs for Cardiovascular Care in Indonesia

**DOI:** 10.1371/journal.pone.0105831

**Published:** 2014-08-22

**Authors:** Asri Maharani, Gindo Tampubolon

**Affiliations:** 1 Faculty of Medicine, University of Brawijaya, Malang, Indonesia; 2 Institute for Social Change, University of Manchester, Manchester, United Kingdom; Aga Khan University, Pakistan

## Abstract

**Background:**

In the past twenty years the heaviest burden of cardiovascular diseases has begun to shift from developed to developing countries. However, little is known about the real needs for cardiovascular care in these countries and how well those needs are being met. This study aims to investigate the prevalence and determinants of unmet needs for cardiovascular care based on objective assessment.

**Methods and Findings:**

Multilevel analysis is used to analyse the determinants of met needs and multilevel multiple imputation is applied to manage missing data. The 2008 Indonesian Family Life Survey (IFLS4) survey is the source of the household data used in this study, while district data is sourced from the Ministry of Health and Ministry of Finance. The data shows that nearly 70% of respondents with moderate to high cardiovascular risk failed to receive cardiovascular care. Higher income, possession of health insurance and residence in urban areas are significantly associated with met needs for cardiovascular care, while health facility density and physician density show no association with them.

**Conclusions:**

The prevalence of unmet needs for cardiovascular care is considerable in Indonesia. Inequality persists as a factor in meeting needs for cardiovascular care as the needs of people with higher incomes and those living in urban areas are more likely to be met. Alleviation of poverty, provision of health care insurance for the poor, and improvement in the quality of healthcare providers are recommended in order to meet this ever-increasing need.

## Introduction

The burden imposed by cardiovascular diseases on the world population, in terms of disability and death, is heavier than that imposed by any other disease. In 2010, 12% of 2,482 million disability-adjusted life years (DALYs) and 30% of 52 million deaths were attributable to cardiovascular diseases [1]. Ominously, the heaviest burden of cardiovascular diseases has begun to shift from developed to developing countries over the last two decades [2], [3]. In developed countries, the percentage of all deaths due to cardiovascular diseases decreased from 48% in 1990 to 43% in 2010, while in the same period it increased from 18% to 25% in developing countries [1].

The increasing social and economic burden of cardiovascular diseases in developing countries is due not only to high morbidity and mortality rates, but also to the relatively early age at onset among largely young and middle-aged populations [3], [4]. A study conducted in 52 countries revealed that the average age of patients with acute myocardial infarction in South Asians was 52 years, as compared with 60 and 65 years in Europeans and North Americans respectively [5]. Such pattern of premature cardiovascular morbidity and mortality is likely to haunt developing countries increasingly in the future since it means the loss of potential years of healthy life as well as a considerable reduction in economic productivity in these countries. It is therefore urgent to reduce the burden of cardiovascular diseases in developing countries. This can be accomplished in part by learning from developed countries' successes in progressively reducing rates of cardiovascular diseases, which has been accomplished by combining effective preventive health care and suitable treatment for acute cardiovascular events [6].

However, interventions that have been so successfully deployed in developed countries to restrain the growth of cardiovascular diseases are still largely absent in the healthcare systems in most developing countries. This is due to the presence of several obstacles [7]. Firstly, developing countries still struggle with inadequately resourced and unevenly distributed healthcare facilities. The densities of hospital beds and health workers are much lower in these countries than in developed countries [8]. Furthermore, the distribution of health workers varies by locality within countries, with the density of health workers being higher in urban areas than in rural areas due to better living standards and higher salaries in the former. Secondly, healthcare systems in developing countries are focused on curative care at the hospital level and are often centred on the use of high-technology equipment in hospitals that provide extensive and expensive treatment for only a small proportion of the population [6]. As a result, a large proportion of people with high cardiovascular risk may remain undiagnosed, and even those diagnosed may face financial barriers to the access of appropriate treatment. These circumstances may increase the burden of cardiovascular diseases in developing countries [6], [9].

But very little is known about the real needs for cardiovascular care in developing countries and how well those needs are being met. To address this, we study the prevalence and determinants of unmet needs of people with cardiovascular risk based on objective assessment in Indonesia. This study contributes to the existing literature in a number of ways. Firstly, it uses a multilevel model, which can handle clustered or grouped data. The multilevel has become increasingly popular in public health research due to the growing interest in the macro- or group-level determinants of health [10], [11]. This model can correctly estimate the observations that cluster at several levels (nested data structure). The use of ordinary regression analysis would be inappropriate given that it neglects the nested structure of the data; failing to take the nested structure in account might lead to an underestimation of standard errors of the higher-level variables. As a result, the significance of such effects is overestimated in such an analysis. The multilevel model, by contrast, accounts for the nested structure of individuals within districts by separating individual variance from district variance. Therefore this model is particularly suited for the present study as the units of analysis in the research are individuals (at a lower level) who are nested within districts (at a higher level). A hypothesis on the associations between district characteristics and individual unmet needs for cardiovascular care (a two-level hypothesis) can thus be tested using this model. Secondly, to avoid self-reporting bias, this study uses objective measurement of cardiovascular risk to measure needs for cardiovascular care using the Reynolds Risk Score [12]. Finally, this study is one of the first to use an up-to-date multi-source dataset in a developing country (Indonesia) in this research area. Specifically, the research questions to be addressed are: What is the prevalence of unmet needs for cardiovascular care services in Indonesia? To what extent do individual determinants such as income, possession of health insurance and residence in urban versus rural areas influence the meeting of needs for cardiovascular care? To what extent do contextual determinants such as health facility density and physician density affect the meeting of needs for cardiovascular care?

The paper answers these questions by using data from Indonesia and by applying a multilevel model, which is geared to finding answers in two-level data such as individuals residing in districts. The paper then presents the results, highlighting inequalities in unmet needs for cardiovascular care along both social and spatial dimensions, that is, across socio-economic groups and across districts. Lastly, it discusses and draws out lessons for public health in anticipating the increasing need for cardiovascular care in developing countries and ways to meet that need. By way of conclusion, the paper lays out future avenues for research that are particularly relevant for the investigation of healthcare needs in developing countries.

## Data and Methods

### Study site

With an estimated population of 234 million in 2008, Indonesia is the fourth-largest country in the world after China, India, and the United States [13]. Indonesia is a lower-middle income country with a per capita GDP of USD $2,178 in 2008 [14]. Less than 3% of the GDP was spent on the health sector in 2006. This percentage is relatively low compared to the average for countries in the East Asia and Pacific region (6.1%) and in the lower-middle income group (5.9%) in the same year [15]. Approximately 36% of this spending is undertaken by public sector, while the rest is undertaken by the private sector. By far the largest source of private spending is direct individual payments made by households, which constitute nearly half of total health expenditures in Indonesia. It is widely accepted that financial protection, especially for the poor, against high levels of self-payments should be one of the primary goals in reforming the health sector given that high levels of self-payments for health can amount to catastrophic spending and lead to impoverishment [16].

Accompanying Indonesia's healthcare financing challenges are healthcare provision challenges. Indonesia's densities of both health facilities and health workers are lower than those of other countries in the region. Indonesia has about 2.5 hospital beds per 10,000 people, whereas other ASEAN countries boast higher averages. For example, Cambodia and Laos offer, respectively, double and triple Indonesia's average number of beds [17]. Health worker density shows a similarly unfavourable comparison. Indonesia has only about 13 public doctors per 100,000 population, such that a doctor must facilitate health services for about 7,600 people who might seek public healthcare [17]. This number places Indonesia far behind the Philippines, a country whose per capita income is similar to that of Indonesia and in which 53 public doctors are available per 100,000 population.

**Figure 1:**
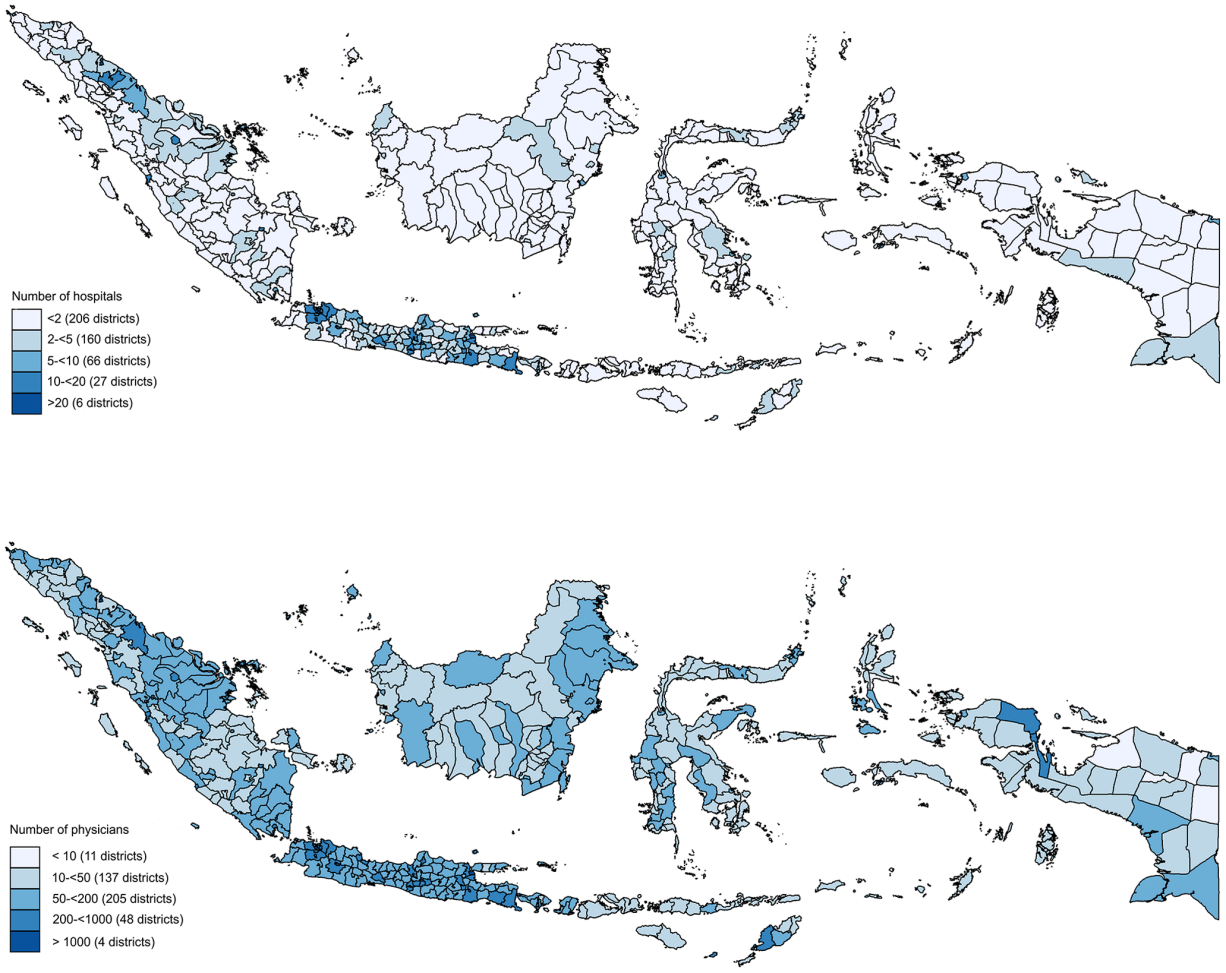
Number of hospitals and physicians across district in Indonesia 2008.

Not only there are too few hospitals and physicians in Indonesia, but they are also inequitably distributed across the archipelago. Figure 1 highlights this inequitable distribution, illustrating that nearly half of Indonesia's districts (206 districts) have only one hospital, while 6 districts have more than 20 hospitals. A similar disparity also occurs in the number of physicians across districts. Eleven districts have more than 1,000 physicians while more than one hundred districts have fewer than 50 physicians and eleven districts have fewer than 10 physicians. This wide gap of supply across districts necessitates analysis at the district and individual levels, not at the national level.

The health financing and provision challenges discussed above will soon become major issues given that the needs for non-communicable disease treatment are expanding rapidly. Like other developing countries, Indonesia is undergoing an epidemiological transition with a rising burden of non-communicable diseases, especially cardiovascular diseases. Cardiovascular disease is one of the main causes of death in Indonesia, accounting for 31.9% of all deaths in the country and 11.2% of all deaths in hospitals [18]. Risk factors such as smoking, poor diet and lack of exercise are growing in importance and further contributing to the incidence of these diseases. This adds urgency to the task of evaluating the needs for cardiovascular care in Indonesia, determining to what degree those needs are currently being met, and discovering how the individual and district characteristics correlate with the needs that remain unmet. To assess these factors, we use data sourced from multiple surveys (individual and contextual) in 176 districts.

### Data

This study combines data from various sources. We use data from the fourth round of the Indonesian Family Life Survey (IFLS4), which was carried out in 2007–2008 by a team from the RAND Corporation in conjunction with Indonesian researchers and various international agencies. The IFLS4 is the main source for individual-level data. It provides individual information on health status (based on blood tests and blood pressure), cardiovascular care utilisation, demographic status and socio-economic status. Alongside IFLS4, we assemble district info from the Indonesian Ministry of Health and Ministry of Finance.

The IFLS4 data has been linked to the other data sources and fiscal statistics using district codes. Firstly, it is linked to health provision data taken from Podes in the same year (2007). The Podes dataset consists of detailed information about the infrastructure of an area, including the number of health facilities and physicians in each village within a district, the aggregate of which is calculated for each district. Next, we link it to other data, including district government fiscal data from the Ministry of Finance. Taken together this data captures the nested structure of individuals within districts. The aim of combining the data in this way is to enhance the specification of models, to reduce confounding and to reduce the likelihood of errors in measurement.

### The dependent variable: unmet demand for cardiovascular care

We calculate cardiovascular risk using the Reynolds Risk Score [19], [20]. This score has been used for cardiovascular disease prevention in several developed countries including the United States and Canada [21], [22]. Previous research shows that the Reynolds Risk Score predicts cardiovascular risk better than the Framingham Risk Score, especially in women [23], [24]. The Reynolds Risk Score is calculated using different formulae for males and females based on age, systolic blood pressure, smoking behaviour, parental history of premature myocardial infarction, and levels of several biomarkers: total cholesterol, high-density lipoprotein (HDL) cholesterol and C-reactive protein.

The computational formula for 10-year cardiovascular risk in men is as follows [20]:
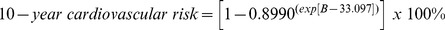
where:

B = 4.385× natural logarithm (age) +2.607× natural logarithm (systolic blood pressure) +0.180× natural logarithm (high-sensitivity C-reactive protein) +0.963× natural logarithm (total cholesterol) –0.772× natural logarithm (high-density lipoprotein cholesterol) +0.818 (if current smoker) +0.438 (if family history premature myocardial infarction)

The computational formula for 10-year cardiovascular risk in women is as follows [19]:
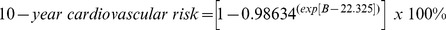
where:

B = 0.0799 (age) +3.137× natural logarithm (systolic blood pressure) +0.102× natural logarithm (high-sensitivity C-reactive protein) +1.382× natural logarithm (total cholesterol) – 1.172× natural logarithm (high-density lipoprotein cholesterol) +0.314× haemoglobin A_1c_ (if diabetic) +0.405 (if current smoker) +0.541 (if family history premature myocardial infarction).

No study has used the Reynolds Risk Score to calculate the cardiovascular risk of individuals in developing countries [25]; this is primarily mostly due to the unavailability of biomarker data. Although cholesterol levels are increasingly available in population-based epidemiological and biomedical surveys, C-reactive protein levels are rarely measured. This study is among the first to use C-reactive protein levels in developing countries measured using Dried Blood Spot C-reactive protein (DBS-CRP). The DBS-CRP is useful in data collection on a sprawling archipelago like Indonesia because it is relatively easy to collect and because samples do not need to be processed and frozen immediately. The validation and quality control processes of the DBS-CRP are carried out before data collection [26]. The limitation of this procedure is the unavailability of data on blood glucose and haemoglobin A_1c_ for women.

We include only respondents aged 40 years or over since blood test results are available only for those in that age category. We classify respondents with 5% and higher 10-year risk as being at risk for cardiovascular events, which in turn indicates that they are in need of cardiovascular care [19]. The levels of unmet needs are arrived at via comparisons of data indicating extant cardiovascular risk and use of cardiovascular care. We measure cardiovascular care utilisation based on the respondents' were having been diagnosed as having heart disease, stroke or hypertension by health workers or having had specific medical examinations for cardiovascular diseases: electrocardiogram examinations or blood tests.

### Covariates

We use the socio-economic and demographic status of individuals as determinants at the individual level. Per capita expenditure, rather than income, is used as a measure of economic status as is common in studies set in developing countries [27], [28], [29]. The per capita expenditure variable is entered as a log-transformed continuous variable to make the distribution more symmetric and to reduce the effect of outliers. Household size measures the total number of household members. Other covariates at the individual level are entered as dummy variables, e.g. marital status (1: married; 0: unmarried), education (1: secondary school or higher; 0: primary school or less), possession of health insurance (1: having health insurance; 0: having no health insurance), and residential location (1: living in a rural area; 0: living in a urban area).

A number of determinants measuring variations in health provisions are used to examine the supply side of healthcare at the district level. The densities of health facilities and of physicians are used to measure the availability of healthcare providers, especially in regard to cardiovascular care. Indonesia has several types of health facilities: hospitals, private clinics, public health centres (*puskesmas*), and sub-health centres (*puskesmas pembantu*). We exclude health centres and sub-health centres because they are not required to provide cardiovascular care services [30]. We also use per capita gross domestic product (GDP) in each district as a district-level determinant. Similar to our treatment of per capita expenditure, we enter per capita GDP as a log-transformed continuous variable in the models.

### Methods

Individuals' met needs for cardiovascular care are calculated as a function of individual and district factors: 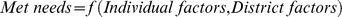
where individual-level variables include marital status, education, household size, residential location, and per capita expenditure and where district-level variables include health facility density, physician density, and per capita GDP.

This research uses multilevel models in order to consider the nested structure of data gleaned from individuals within districts. Considering individual i nested in districts j, the model is:
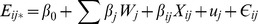
with:

E_ij*_ = logit (*P* (E_ij*_ = 1))

*W_j_* is a set of district characteristics,

*X_ij_* is a set of individual characteristics,

*u_j_* are the random intercept varying over district with mean zero and variance σ_u_^2^


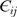
 is normally distributed with mean zero and variance 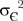.

### Missing data

Missing data appears at the individual levels. To avoid potential bias due to mishandling of incomplete data, we apply multilevel multiple imputation [31], [32]. We present robust analysis by analysing the original data and the multiply imputed data and by comparing the results (see Appendix S1).

## Results

**Figure 2:**
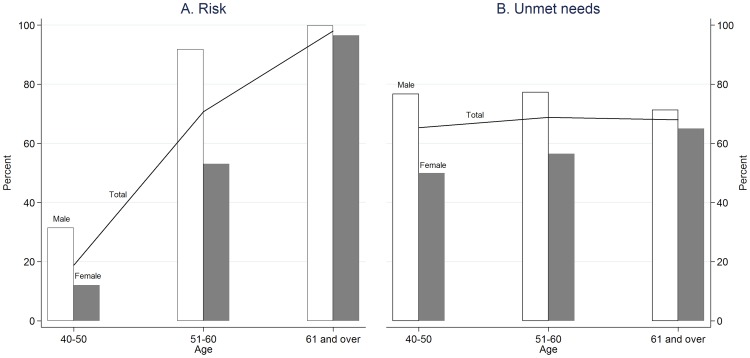
Percentages of at risk and unmet needs for cardiovascular care among men and women.

A total of 3,406 respondents aged 40 years and over were initially included. About 60% of respondents were women and 35% of respondents were 60 years old and over. Figure 2 shows that, overall, the proportion of respondents at risk for a cardiovascular event increases with age. However, the risk patterns differ between men and women. The proportion of men at cardiovascular risk increases dramatically with age up to 60 years and then continues to rise only slightly, while that of women increases considerably with age. Only one-third of respondents at risk receive the needed treatment. The proportion of male respondents with unmet needs is higher than that of women. Like the patterns of cardiovascular risk proportions, the proportion of unmet needs differs between men and women. The proportion of men with unmet needs increases dramatically up to 60 years old and then declines, while that of women continues to increase with age.

**Figure 3:**
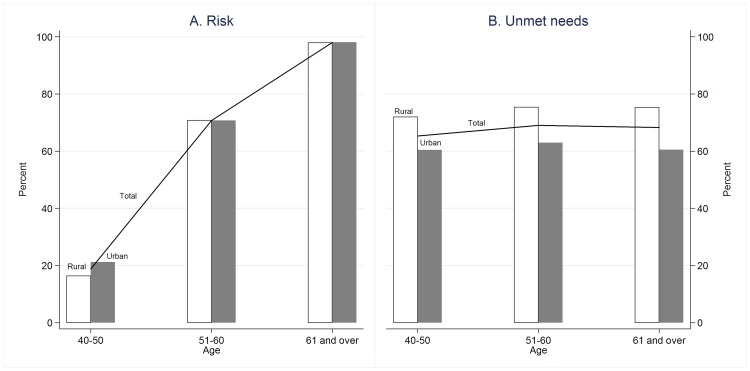
Percentages of at risk and unmet needs for cardiovascular care in rural and urban areas.

Figure 3 compares the prevalence of cardiovascular risk and unmet needs for cardiovascular care in urban areas with that in rural areas. The figure shows that the prevalence of cardiovascular risk among rural residents aged less than 50 years old is lower than that among urban residents in the same age group, while the prevalence of the risk among elderly people in rural and urban areas is equal. Compared to their urban counterparts, fewer rural residents receive cardiovascular services when they need them. The prevalence of unmet needs both in rural and urban areas remains constant across age groups.

For the analysis of determinants of unmet needs, we include respondents with cardiovascular risk only. The descriptive statistic of these respondents (see Table 1) shows that the majority of respondents in this category were married (73.24%) and did not attend junior high school or other higher education programmes (65.81%). Only 28.31% of the respondents had health insurance and just over half of respondents lived in rural areas (50.16%). On average, each household consisted of 4.05 individuals (SD = 2.17) and the average monthly per capita household expenditure was IDR 537,890 or approximately USD $55.82 at the historical 2007 exchange rate [33]. There is a considerable amount of missing data in the education variable (26%); we optimised the utilisation of this invaluable cardiovascular risk data by imputing the missing data [31], [32].

**Table 1:** Sample characteristics and explanatory bivariate analysis (2,249 individuals in 176 districts).

	Mean ± SD (%)	Met needs	Marginal effect
*Individual characteristics*			
**Marital status:**			
Married	73.3	30.8	−0.033
Unmarried	26.7	34.1	0.000
**Education:**			
Primary school or less	65.8	28.6	0.000
Secondary school or more	34.2	40.8	0.096^‡^
**Health insurance:**			
Have health insurance	28.3	38.7	0.095^‡^
Have no health insurance	71.7	28.9	0.000
**Residential location:**			
Rural	50.2	24.8	−0.135^‡^
Urban	49.8	38.5	0.000
**Household size**	4±2.2		−0.0001
**Per capita expenditure (IDR 1,000)**	537.8±557.8		0.107^‡^
*District characteristics*			
**Health facilities density (in 10,000 population)**	0.9±0.9		0.009^‡^
**Physician density (in 100,000 population)**	2.6±2.5		0.037^‡^
**Per capita GDP (IDR 1,000)**	19,703±36,397		0.064^‡^

Sig.: ^‡^significant at 1% or less.

The bivariate logistic regression model (see Table 1, fourth column) shows that all but two predictors are statistically significant with met needs at the 1% level. At the individual level, being relatively well educated, having health insurance and having a higher level of per capita expenditure all correlate positively with met needs, whereas living in a rural area shows an inverse relationship. Being married and living in a larger household are elements that both fail to achieve statistical significance, but they correlate with lower levels of met needs. At the district level, higher levels of health facility density, physician density and per capita GDP have significant and positive correlations with met needs.

These bivariate correlations point to complex patterns of associations between met needs for cardiovascular care and the risk factors. To avoid confounding relationships and to arrive at net associations, results from three multilevel logistic models are presented next: Model 1 excludes supply side factors while Models 2 and 3 include supply side factors (Table 2). The results show that being married has a significant and negative association with met needs, while education and household size have no significant correlation with met needs. Those who have health insurance are more likely to get cardiovascular care when they need it. The needs of respondents with higher expenditure levels are more likely to be met, while respondents living in rural areas have a 38% lower probability of experiencing met needs. These statistically significant results for economic status and area of residence suggest that there is inequality in meeting the needs for cardiovascular care in Indonesia.

**Table 2:** Determinants of met needs.

	Model 1	Model 2	Model 3
Married	−0.34(0.14)^†^	−0.33(0.14)^†^	−0.33(0.14)^†^
Secondary school and higher	0.02(0.13)	0.01(0.13)	0.01(0.13)
Have health insurance	0.37(0.12)^‡^	0.38(0.12)^‡^	0.38(0.12)^‡^
Household size	0.02(0.03)	0.02(0.03)	0.02(0.03)
Log per capita expenditure	0.36(0.09)^‡^	0.35(0.09)^‡^	0.36(0.09)^‡^
Rural	−0,38(0.14)^‡^	−0.34(0.14)^‡^	−0.34(0.15)^‡^
Health facilities density (in 10,000 population)		0.01(0.01)	
Physician density (in 100,000 population)			0.02(0.06)
Log per capita GDP		0.06(0.10)	0.08(0.10)
Between district variance	0.23	0.23	0.22
ICC	0.06	0.06	0.06
Median odds ratio	1.58	1.57	1.57

Reported are marginal effects (standard error).

Sig.: ^†^significant at 5% or less; ^‡^significant at 1% or less.

We include supply side factors in both the second and third models. The supply side factor in Model 2 is the density of health facilities; that in Model 3 is the density of physicians. We use two different models to avoid multicollinearity since health facility density and physician density are highly correlated (0.76). The results indicate that no determinant at the district level has a significant association with met needs. The high median odds ratio shows that the disparities in meeting cardiovascular care needs persist not only at the individual level, but also at the district level. Furthermore, we compare the results of analysis using multilevel multiple imputed data and those excluding all individuals with missing values to check the robustness the results (see Appendix S1). The no substantial differences between those two results indicate that the results are robust.

**Figure 4:**
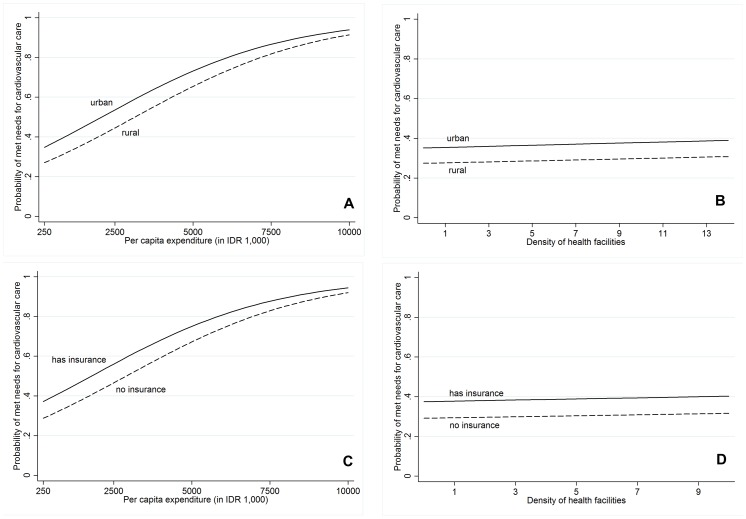
Predicted probability of met needs for (A) residential location and per capita expenditure, (B) residential location and density of health facilities, (C) health insurance and per capita expenditure, and (D) health insurance and density of health facilities.

For ease of understanding, we plot the predicted probability of met needs as a function of per capita expenditure (representing economic status) with separate curves for residential areas and health insurance possession in Figure 4a and Figure 4c respectively. The results show that increased per capita expenditure is associated with an increased likelihood of cardiovascular care needs being met, suggesting that inequalities persist in terms of access to such care. Focusing on residential areas, there is a gap between people living in urban areas and those living in rural areas with the same per capita expenditure, confirming that living in urban areas increases the probability of met needs. However, the gap slightly narrows after per capita expenditure achieves IDR 7,500,000, which implies that the probability of obtaining cardiovascular care converges for the relatively well-off regardless of whether they live in rural or urban areas. The similar pattern in Figure 4c also highlights the effect of economic status on met needs. Beyond a certain level of expenditure, people with and without health insurance coverage have converging probabilities of cardiovascular care needs being met.

The predicted probability of met needs as a function of health facility density with separate curves for respondents living in urban and rural areas and for respondents having health insurance and having no health insurance are shown in Figure 4b and 4d, respectively. These figures show that the presence of a greater number of health facilities only marginally improves the probability of met needs for cardiovascular care. Respondents who live in urban areas (Figure 4b) and those who possess health insurance (Figure 4d) have a higher probability of met needs among populations with the same density of health facilities.

## Discussion

Although the burden of cardiovascular diseases is increasing sharply in developing countries, there is a paucity of data revealing the real needs for cardiovascular care in these countries and the extent to which those needs are currently being met. This study, therefore, investigates the prevalence and determinants of unmet needs of cardiovascular care in Indonesia using a new national sample. The results show that the proportion of people with unmet needs for cardiovascular care is considerable. Indonesia fails to provide its population with sufficient cardiovascular care, as indicated by the fact that only one-third of survey respondents with cardiovascular risk were receiving cardiovascular care at the time of the study. Because both monitoring and effective treatment of cardiovascular diseases are currently lacking, Indonesia may experience a higher burden due to substantial morbidity, mortality, and increasing healthcare costs in the decade ahead. The identification of factors that distinguish those with met needs for cardiovascular care from the rest of the population may suggest how the current levels of unmet needs can be reduced, thus staving off the tremendous anticipated burden.

At the individual level, higher levels of per capita expenditure, possession of health insurance and residence in a rural area are important determinants in explaining discrepancies in met needs for cardiovascular care. Individuals who are better off have a higher likelihood of receiving cardiovascular care services even if they live in rural areas or have no health insurance (see Figure 4). This underlines the fact that poverty increases the risk of having unmet healthcare needs; see also [34], [35], [36]. There are several explanations for this finding. Firstly, higher income is associated with more frequent and more intensive use of health services in developing countries [37]. In Indonesia, the poor use inpatient service in hospitals 60% less than the relatively wealthy [17]. Secondly, people with higher incomes tend to seek care from modern health care providers rather than traditional practitioners [37]. With a considerable number of traditional healers and unlicensed drug sellers in urban and rural poor communities, almost half of Indonesia's population rely on self-treatment when they are ill [15]. Finally, the failure of health services to reach the poor in developing countries is partly due to the fact that the poor receive less government subsidies in the health sector than those who are better off [38]. Indonesia spends about 40% of public healthcare resources on regressively targeted subsidies to public hospitals. Of the funding that is spent on hospital care, only 13% benefits the poorest quintile of the population, while about 34% benefits the richest quintile [17]. Public spending generally benefits higher-income groups since the poor use hospital services less than those who are better off. In addition, the poor incur additional costs when accessing hospital care, including transportation costs and opportunity costs, which further deter them from using hospital services to meet their needs.

The positive correlation of poor household economic conditions with unmet needs may highlight the role of health insurance in protecting the population, especially the poor. The present study finds that respondents who have health insurance are more likely to receive cardiovascular care when they need it. This finding confirms previous studies in the United States showing that uninsured adults are significantly more likely than insured adults to have unmet needs for preventive services [39], [40]. In Indonesia, however, the role of health insurance in reducing the levels of unmet needs for cardiovascular care, especially among the poor, still needs to be evaluated. Notwithstanding the success of *Askeskin*, the health insurance scheme intended to decrease out-of-pocket spending among the poor [41], the utilisation of *Askeskin* at the hospital level is very low. Only 8.5% of *Askeskin* holders use hospital services, while more than 90% use it only in health centres or sub-health centres and not for hospital services [17]. Since cardiovascular services are available mostly in hospitals, which can only be accessed with additional costs (for instance, transportation cost), the needs of the poor for these services may remain unfulfilled.

Turning to the variable of residential areas, respondents who live in rural areas have a lower probability of having met needs for cardiovascular care, a finding consistent with previously published work [42]. The characteristics of rural and urban areas vary significantly [43] and access to healthcare may therefore operate differently in rural and urban areas. In Indonesia, the number of physicians per 100,000 residents in urban areas is at least six times that in rural areas. In 2007, urban areas had 36 physicians per 100,000 residents, while rural areas had only 6 per 100,000 residents [44]. The gap in meeting healthcare needs between rural and urban areas is not only due to the supply of healthcare providers, but also to the paucity of social and economic infrastructure in rural areas [45]. For example, people in rural areas have higher proportions of poorly educated household heads, lack of sanitation and lack of access to clean water compared to those living in urban areas [46].

At the district level, this study finds that neither the density of health facilities nor the density of physicians varies significantly with met needs for cardiovascular care. To our knowledge, there is no prior evidence of such correlation in developing countries; prior evidence collected in developed countries has, however, yielded mixed results. Two studies in the United States revealed no association between supply of health care resources and unmet health needs [42], [47], while another study from the same country concluded that higher densities of paediatricians are associated with a lower prevalence of perforated appendicitis. A study in South Korea found a positive and significant relationship between the number of regional hospital beds in the private sector and unmet needs for healthcare but no associative relationship between density of public health providers and unmet needs [36]. Since developing and developed countries have different conditions with respect to health provisions, our findings prompt careful consideration. Lack of association between health provision and unmet needs for cardiovascular care in the current study should not be taken as evidence that numbers of health facilities and physicians are unimportant, since availability of healthcare providers often emerges as an important determinant of service utilisation and health outcomes in developing countries [44]. Our findings may indicate the presence of other covariates creating barriers to cardiovascular care utilisation despite a relatively high density of health providers. For example, the poor people without health insurance have a lower probability of met needs than those with health insurance in the same districts (see Figure 4d), implying that the high cost of cardiovascular care may be prohibitive for the poor without health insurance. Another plausible explanation is that the overall level of health providers' diagnostic and treatment abilities is low in Indonesia. The average scores for the quality of adult curative care provided by public and private healthcare workers in Indonesia are 49% and 56% out of 100%, respectively [44].

This study has a number of limitations; future work may be motivated by a wish to overcome them. Firstly, although it has demonstrated a positive association between per capita expenditure and met needs for cardiovascular care, this study has not provided an estimate of its causal effect. The estimation of the causal effect of per capita expenditure on unmet needs using observational data requires researchers both to solve the reverse causality problem and to control for all other unobserved factors. Future research may employ an instrumental variable estimator so that reverse causality can be ruled out while simultaneously controlling for at least all time-constant unobservable determinants. Secondly, the data used for this study was not originally designed for use in calculating the Reynolds Risk Score. The missing data on blood glucose and haemoglobin A_1c_ may affect the calculations as it prevents us from identifying women with diabetes mellitus; we are therefore unable to calculate the score of haemoglobin A_1c_ among diabetic women. Therefore, the proportion of women at risk might well be greater than indicated by our calculation if the data were available and taken into account. Thus these estimates can be taken as lower bounds of the proportion of women at risk. Additional data collection is needed to improve the accuracy of this portion of the results. Finally, there is as yet no cardiovascular risk test designed specifically for residents of developing countries. The designing of such a test is urgently needed.

Despite these limitations, our findings have several important implications for policy makers. Firstly, in following previous research, the present study provides an additional source of empirical support for generally established view regarding the positive associations between poverty and unmet needs for cardiovascular care. Policy makers may now wish to explore poverty alleviation efforts more closely, particularly in the context of the considerable impact such efforts can have on meeting the population's needs for cardiovascular care. Secondly, policy makers should provide the population with an effective healthcare insurance scheme. The plan of the Indonesian government to achieve universal coverage in 2019 should be monitored and evaluated to ensure that all Indonesians, especially the poor, receive appropriate healthcare services. Finally, this study makes it clear that merely increasing the number of health facilities available has not improved utilisation or delivery of cardiovascular care. Meeting the population's needs for cardiovascular care will demand consideration of the quality of the healthcare provided, especially with regard to the diagnostic and treatment abilities of practitioners.

We conclude that the prevalence of unmet needs for cardiovascular care is considerable in Indonesia, resulting in projections of high morbidity and mortality in the decades ahead. Deep inequality persists in the provision of access to needed cardiovascular care services: the majority, i.e. the poor and those living in rural areas, have a lower probability of accessing these services as needed. The alleviation of poverty, the provision of effective healthcare insurance, and improvements in the quality of healthcare providers are all recommended in order to improve access to prevention, early diagnosis, and early treatment for populations with cardiovascular risks. This will result in reduced morbidity and mortality as well as lower levels of hospitalisation due to cardiovascular diseases, which in turn will increase the economic productivity of the country.

## Supporting Information

Appendix S1 **Determinants of met needs: before and after multiple imputation.**
(DOCX)

Appendix S2 **Correlation between variables**
(DOCX)
