# Supplementary material for: Unmet Needs for Cardiovascular Care in Indonesia
Source: PLoS One. 2014 Aug 22;9(8):e105831. doi: 10.1371/journal.pone.0105831 (PMC4141811; doi:10.1371/journal.pone.0105831)
Supplement: Appendix S1 — Determinants of met needs: before and after multiple imputation. (DOCX) [file pone.0105831.s001.docx]

**Appendix S1. Determinants of met needs: before and after multiple imputation**

|  | **Model 1** | **Model 2** | **Model 3** | **Model 1** | **Model 2** | **Model 3** |
| --- | --- | --- | --- | --- | --- | --- |
| Intercept | 0.00(0.01)‡ | 0.01(0.01)‡ | 0.01(0.01)‡ | 0.00(0.00)‡ | 0.00(0.00) ‡ | 0.00(0.00) ‡ |
| Married | 0.71(0.10)* | 0.71(0.10)* | 0.71(0.10)* | 0.82(0.09)* | 0.82(0.09)* | 0.82(0.09)* |
| Secondary school and higher | 1.02(0.14) | 1.01(0.13) | 1.01(0.13) | 0.96(0.12) | 0.95(0.12) | 0.95(0.12) |
| Have health insurance | 1.45(0.18)‡ | 1.46(0.18)‡ | 1.46(0.18)‡ | 1.44(0.16) ‡ | 1.44(0.16) ‡ | 1.44(0.16) ‡ |
| Household size | 1.02(0.03) | 1.02(0.03) | 1.02(0.03) | 1.02(0.02) | 1.01(0.02) | 1.01(0.02) |
| Log per capita expenditure | 1.44(0.14)‡ | 1.42(0.14)‡ | 1.42(0.14)‡ | 1.47(0.12) ‡ | 1.45(0.12) ‡ | 1.45(0.12) ‡ |
| Rural | 0,68(0.09)‡ | 0.71(0.10)‡ | 0.71(0.10)‡ | 0.64(0.08) ‡ | 0.69(0.09) ‡ | 0.68(0.09) ‡ |
| Health facilities density (in 10,000 population) |  | 1.01(0.01) |  |  | 1.01(0.01) |  |
| Physician density (in 100,000 population) |  |  | 1.02(0.06) |  |  | 1.02(0.05) |
| Log per capita GDP |  | 1.06(0.11) | 1.08(0.11) |  | 1.09(0.10) | 1.13(0.11) |
| Between district variance | 0.23 | 0.23 | 0.22 | 0.23 | 0.22 | 0.22 |
| ICC | 0.06 | 0.06 | 0.06 | 0.06 | 0.06 | 0.06 |
| Median odds ratio | 1.58 | 1.57 | 1.57 | 1.58 | 1.56 | 1.56 |

Reported are odds ratio (standard error).

Sig.: †: significant at 5% or less; ‡: significant at 1% or less
