# Supplementary material for: Unmet Needs for Cardiovascular Care in Indonesia
Source: PLoS One. 2014 Aug 22;9(8):e105831. doi: 10.1371/journal.pone.0105831 (PMC4141811; doi:10.1371/journal.pone.0105831)
Supplement: Appendix S2 — Correlation between variables (DOCX) [file pone.0105831.s002.docx]

**Appendix S2. Correlation between variables**

|  | Met needs | Married | Secondary school or more | Have health insurance | Household size | Log per capita expenditure | Rural | Hospital density (in 10,000 population) | Physician density (in 100,000 population) | Log per capita GDP |
| --- | --- | --- | --- | --- | --- | --- | --- | --- | --- | --- |
| Met needs | 1.0000 |  |  |  |  |  |  |  |  |  |
| Married | -0.0318 | 1.0000 |  |  |  |  |  |  |  |  |
| Secondary school or more | 0.1140 | 0.1619 | 1.0000 |  |  |  |  |  |  |  |
| Have health insurance | 0.0955 | 0.0368 | 0.2396 | 1.0000 |  |  |  |  |  |  |
| Household size | -0.0006 | 0.1355 | 0.0519 | 0.0035 | 1.0000 |  |  |  |  |  |
| Log per capita expenditure | 0.1643 | 0.0151 | 0.3773 | 0.1175 | -0.2334 | 1.0000 |  |  |  |  |
| Rural | -0.1468 | -0.0017 | -0.3235 | -0.1296 | -0.0710 | -0.2892 | 1.0000 |  |  |  |
| Hospital density (in 10,000 population) | 0.0979 | -0.0069 | 0.2406 | 0.1271 | 0.0649 | 0.1948 | -0.4217 | 1.0000 |  |  |
| Physician density (in 100,000 population) | 0.1279 | 0.0062 | 0.2821 | 0.1249 | 0.0903 | 0.2760 | -0.5350 | 0.7172 | 1.0000 |  |
| Log per capita GDP | 0.1188 | 0.0191 | 0.1833 | 0.0643 | 0.0938 | 0.2532 | -0.3132 | 0.3912 | 0.5603 | 1.0000 |
